# Highly Arid Oasis Yield, Soil Mineral N Accumulation and N Balance in a Wheat-Cotton Rotation with Drip Irrigation and Mulching Film Management

**DOI:** 10.1371/journal.pone.0165404

**Published:** 2016-10-31

**Authors:** Jinling Lv, Hua Liu, Xihe Wang, Kaihui Li, Changyan Tian, Xuejun Liu

**Affiliations:** 1 Xinjiang Institute of Ecology and Geography, Chinese Academy of Sciences, Urumqi 830011, China; 2 College of Resources and Environmental Sciences, China Agricultural University, Beijing 100193, China; 3 Institute of Plant Nutrition, Resources and Environmental Sciences, Henan Academy of Agricultural Sciences, Zhengzhou, China; 4 Soil and Fertilizer Institute, Xinjiang Academy of Agricultural Sciences, Urumqi 830000, China; National Key Laboratory of Crop Genetic Improvement, CHINA

## Abstract

Few systematic studies have been carried out on integrated N balance in extremely arid oasis agricultural areas. A two-year field experiment was conducted to evaluate the N input and output balances under long-term fertilization conditions. Five treatments were chosen, namely CK (no fertilizer), NPK, NPKS (10% straw return N and 90% chemical N), NPKM (one third urea-N, two thirds sheep manure) and NPKM+ (1.5 times NPKM). The results show an abundance of dry and wet N deposition (33 kg N ha^-1^ yr^-1^) in this area. All treatments (excluding CK) showed no significant difference in wheat production (P>0.05). NPKM gave higher cotton yields (P<0.05). In both crops, NPKM and NPKS treatments had a relatively higher N harvest index (NHI). ^15^N-labeled results reveal that the fertilizer N in all N treatments leached to<1 m depth and a high proportion of fertilizer-N remained in the top 60 cm of the soil profile. The NPKM+ treatment had the highest residual soil mineral N (N_min_, 558 kg Nd ha^-1^), and NPKM and NPKS treatments had relatively low soil N_min_ values (275 and 293 kg N ha^-1^, respectively). Most of the treatments exhibited very high apparent N losses, especially the NPKM+ treatment (369kg N ha^-1^). Our arid research area had a strikingly high N loss compared to less arid agricultural areas. Nitrogen inputs therefore need careful reconsideration, especially the initial soil Nmin, fertilizer N inputs, dry and wet deposition, and appropriate organic and straw inputs which are all factors that must be taken into account under very arid conditions.

## Introduction

Most nitrogen (N) pollution results from human activities [1] and especially those linked to the use of N fertilizers in intensive agriculture [2–4]. Nitrogen use efficiency (NUE) in Chinese agriculture can be as low as 26–36% (e.g. only 34.8% in wheat and 29.1% in maize) [5]. Overuse of fertilizer N can lead to a number of environmental problems such as groundwater pollution and eutrophication of streams and rivers (nitrate leaching), eutrophication of terrestrial ecosystems (NH_3_ deposition), and contribution to climate change (NO and N_2_O emissions) [6–8]. An increase in NUE and a simultaneous decrease in environmental damage are therefore urgent priorities for agricultural production [9].

The question therefore arises of how to reduce N loss and increase N use efficiency. The most important step is to determine the appropriate N input and output. Nitrogen input largely comprises fertilizers, organic matter, soil mineralization, atmospheric wet and dry deposition and biological N_2_ fixation. Nitrogen output is mainly composed of crop recovery, soil residues, leaching and volatilization [10–14]. However, the N inputs are different under different environmental conditions due to interactions among environmental factors affecting N cycle processes including the mineralization of soil organic carbon (SOC), crop growth, cropping pattern, and soil microbial activity. In addition, changes in agricultural management practice can also significantly change the N inputs and affect the environment by changing NUE and N transport [10,15,16].

Extremely arid desert farmland areas have distinct climatic characteristics and planting patterns including one crop per year which results in oasis desert croplands having longer fallow seasons. Furthermore, large differences between day and night air temperatures affect SOC mineralization and long-term alternative freezing and thawing cycles also affect SOC mineralization and N_2_O emissions [17]. In addition, atmospheric N deposition and irrigation N inputs can also be important sources of N [18]. For example, wet and dry N deposition to farmlands in the oasis area around Urumqi in northwest China can reach 37kg ha^-1^ yr^-1^[19]. More irrigation water is used in arid cropland areas due to the high rates of evaporation (up to 2600 mm yr^-1^ in our research area) and a lack of precipitation (average annual rainfall of 194 mm). The concentration of groundwater nitrate N in our research shows a relatively high value (8mg N L^-1^ on average) well above the average level and this contributes to high soil nitrate N pollution in western China.

Moreover, drip irrigation and plastic film mulching are very prevalent for water conservation in extremely arid oasis areas. According to the Chinese Ministry of Agriculture [20], the mulching cultivation area of China has reached 23 million ha, much of which is accounted for by arid and extremely arid areas. Taking our research area (Xinjiang Autonomous Region, northwest China) as an example, 75% of the cropland (> 3.13 million ha) is covered by mulching film, accounting for 13.6% of the whole of China [21]. Film mulching and drip irrigation can improve the water and N use efficiencies minimizing N leaching is a major objective involved in the use of drip-irrigation and mulching[22–25]. Some studies have demonstrated that film mulching and drip irrigation can reduce ammonia volatilization, increasing the N concentration on the soil surface and thus changing the N cycle[4, 26]. However, a considerable amount of research shows that mulching film can accelerate the mineralization of SOM (soil organic matter) and release more mineralized N into the soil due to increased soil temperature [15,27]. Nitrogen cycling in extremely arid desert croplands therefore has unique characteristics that make it distinct from other, non-arid, areas.

Although there have been some N balance studies in extremely arid areas, most of the work is based on experiments conducted in different fields using only chemical fertilizers or ignoring integrated N balance[23,28]. Few studies have combined environmental N (wet and dry deposition and irrigated N), mineral N and organic and inorganic fertilizer N inputs to access the N input and output balance based on long-term studies. The objectives of the current two-year field study were therefore(1) to determine the amount of N input from N fertilizers, organic matter, soil mineralization, and atmospheric wet and dry deposition in a cotton-wheat rotation system, (2) to understand the impact of different fertilization treatments on wheat and cotton production and N harvest indices and the efficiency of recovery of urea-N using ^15^N stable isotope techniques, and (3) to evaluate the N input and output balance and measure the apparent N loss under drip irrigation and film mulching.

## Materials and Methods

### Experimental site

The N input and output balance experiment was carried out using selected treatment plots of an existing long-term field experiment at the Xinjiang National Grey Desert Soil Station (43° 56′ N, 87° 28′ E) of the Chinese Academy of Agricultural Sciences located on the alluvial plain of the Tian shan Mountains. The study site is typical oasis farmland with a wheat-cotton crop rotation. Mean annual precipitation is 310mm, 70% of which falls in the winter (from December to February) and summer (from June to August). Evaporation (ET_0_) is about 2570 mm annually. The mean annual temperature is 7.7°C. The annual frost-free period is about 156 d(usually from the middle of April to the middle of September) [28]. The soil is a grey desert soil with percentages of clay, silt and sand fractions in the top 20 cm of the soil profile of 30.3, 52.5 and 17.2%, respectively.

### Field experiment

The field was unmanaged natural land before the start of the long-term experiment in April 1989 with the original soil fertility. The soil in the research area belongs to loess-like alluvial-alluvium soil, which was classified as a durisols in the American Soil Taxonomy[29], and had a relatively high SOC content due to its location on the alluvial plain of the Tian shan Mountains, with an SOC content of 15.2 g C kg^-1^, total N content 0.868 g N kg^-1^, total P 0.667 g P kg^-1^, total K 19.8 g K kg^-1^, available P (Olsen-P) 3.4 mg P kg^-1^,available K of 288 mg K kg^-1^and soil pH (water extracted) 8.1 in top 20 cm of the soil profile. Five typical fertilization treatments were selected from the total of twelve treatments in the field experiment. These were CK (no organic or inorganic fertilizers), NPK (application of urea, calcium superphosphate and potassium sulfate at rates of 245 kg N, 60.3 kg P and 48 kg K ha^-1^ yr^-1^, respectively), NPKM(application of 82 kg N, 22.1 kg P and 16 kg K ha^-1^ yr^-1^ and an additional application of farmyard manure (30 t ha^-1^sheep manure containing~164 kg organic N ha^-1^), NPKM+(1.5 NPKM), NPKS (total NPK nutrient inputs as in the NPKM treatment, N input from crop straw which was completely returned to the soil determined, then the remainder of the N input (according to the standard 245kg N ha^-1^) to be supplemented with urea). Sixty percent of the fertilizer N was applied as basal fertilizer and 40% N was applied as a topdressing. However, the topdressing fertilizer for cotton was split into three applications but the topdressing for wheat was input as one application. The P, K, and organic fertilizers were applied as a single basal application. The organic fertilizer was air-dried sheep manure with 5–7 g N, 4–6 g P_2_O_5_ and 3–6 g K_2_O kg^-1^. The experiment was a randomized block design including with triplicate plots of each treatment. Each plot (rectangle, 466 m^2^) was isolated by cement banks with a depth of 70 cm depth and 10 cm above the soil surface to prevent runoff.

**Fig 1:**
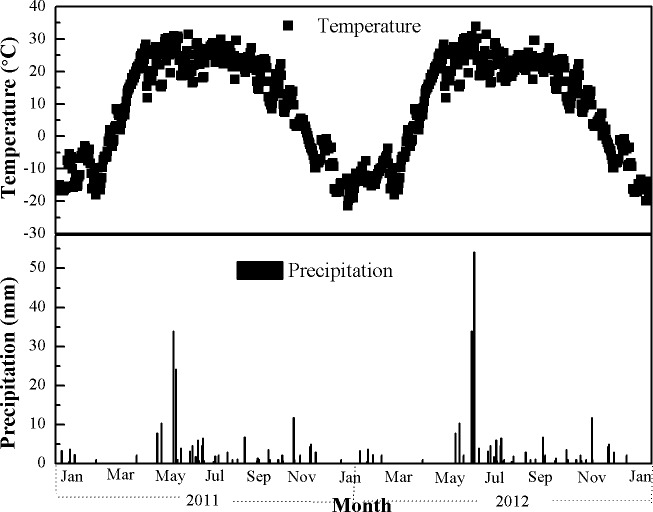
(a) Air temperature and (b) precipitation distribution from 2011 to 2012.

**Fig 2:**
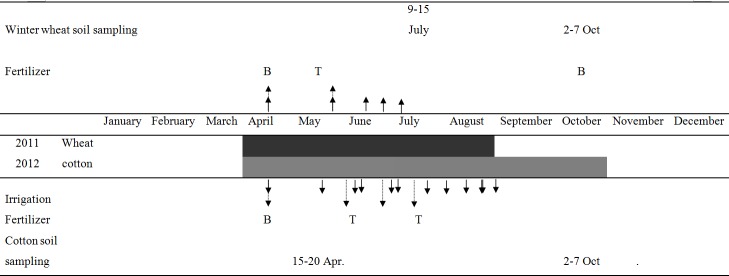
Schedule of planting, fertilizer application, irrigation and harvesting of different crops. (1) Shading indicates that the crop is growing in the field; (2) Solid arrows represent irrigation, dotted arrows represent fertilizer applications; (3) B represents the basal fertilizer and T represents the topdressed fertilizer.

Wheat was sown in April and harvested in the middle of August in 2011. Cotton was sown in late April and harvested in October in 2012. The precipitation and temperature are shown in Fig 1. The drip irritation technique was employed in both the cotton and wheat seasons. The mulching film technique was used only during the cotton season. The drip irrigation method used was the conventional method used in the area, the flow rate was 2-3L h^-1^ and the emitter spacing was around 50cm.The mulch film was polyethylene with a thickness of 0.01–0.02mmand with 1.2m width of application in the cotton season. The field was irrigated five to six times in the wheat season and thirteen to fifteen times in the cotton season depending on natural precipitation levels (Fig 2). The annual average irrigation water was 171 mm for wheat and 363 mm for cotton. Herbicides and pesticides were applied to control weeds and insects, respectively. Wheat and cotton were harvested to the level of the soil surface, leaving negligible stubble in the field according to local practice. All straw was removed from the field but the NPKS treated grain and straw were weighted separately after air drying.

### ^15^N-urea microplot experiment

**Fig 3:**
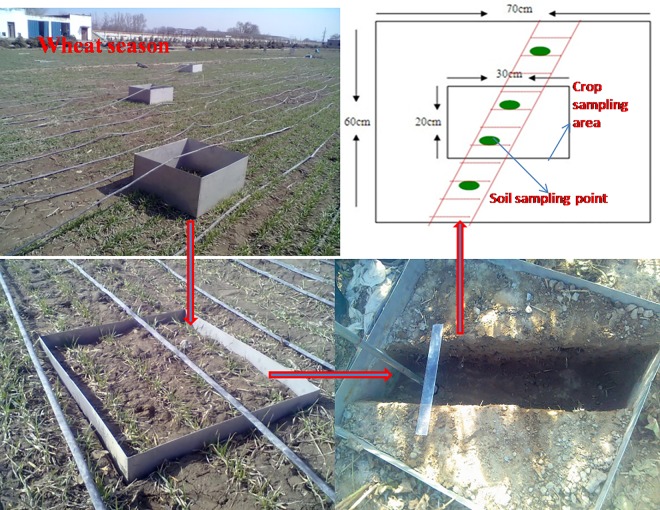
The ^15^N isotope tracing experiment in the wheat season in 2011 (taking the wheat season as an example).

The ^15^N experiment was conducted from mid-April 2011 to October 2012 in order to observe the fate of urea-N in the wheat and cotton seasons under drip irrigation conditions (Fig 3). There were four ^15^N-labeled urea treatments comprising NPKM+, NPK, NPKM and NPKS. Four microplots (0.7×0.6m) were established in the northeast portion of each plot. A syringe was used to inject the urea solution into the microplots to ensure that the crop received an even distribution of added N. Metal squares 0.45 m high were driven 0.40 m deep into the soil to prevent surface runoff and lateral contamination. Urea enriched with 5.2 atom %^15^N (provided by the Institute of Chemical Industry, Shanghai, China) was applied to the soil. All of the P and K applications and field management practices in the microplots were the same as in the corresponding large plots in the wheat and cotton seasons. In all microplots at harvest the soil and fertilizer N recoveries were determined using the following Eqs (1) and (2), where all ^15^N was expressed as the atom % excess corrected for background abundance (0.366%).

$$NdfF_{labeled}in\;soil\;\left(kg\;N\;ha^{-1}\right)=total\;N\;in\;soil\times^{15}N\;atom\;\%\;excess\;in\;plant{/}^{15}N\;atom\;\%\;excess\;in\;fertilizer$$ (1)

The fertilizer N recovery was calculated according by:

FertilizerNrecovery(%)=(totallabeledNintheabovegroundbiomass/labeled−Napplied)×100 (2)

### Sample collection and measurements

Soil samples were collected from all plots and microplots using a 5-cm i.d. auger and separated into 20-cm depth increments from 0 to 1m depth before planting and after the harvest of wheat and cotton (Fig 2). Soil samples were stored in an ice-box immediately after sampling and were then transported to the laboratory for analysis. Within 12 h all the fresh soil samples were extracted with 0.01 mol L^-1^ CaCl_2_ solution (ratio of soil to solution 1:10). The extracts were analyzed for NO_3_^—^N and NH_4_^+^-N as mineral N (N_min_) with an AA3 continuous flow analyzer (Seal Analytical, Southampton, UK). Soil samples from the microplots were air-dried and ground to pass a 0.15mm screen for total N and ^15^N isotope analysis. Irrigation water was also collected, filtered into sealed bottles and stored in an ice-box immediately until analysis.

Both crops were separated into grain and straw after the harvest and calculated the above biomass, grain and straw production. Grain and straw samples were subsequently dried at 105°C in a forced air oven and were ground to pass a 150-μm screen. Grain, straw and soil samples were analyzed for total N and ^15^N abundance by the micro-Kjeldahl procedure and by isotope ratio mass spectrometry (Delta Plus, Thermo Fisher, Waltham, MA).

### The wet and dry deposition experiment

Rainwater samples were collected with precipitation collectors directly after every rainfall event. The rain samples were analyzed for NH_4_^+^ and NO_3_^-^(N_min_) using the AA3 continuous flow analyzer (Seal Analytical, Southampton, UK). Wet deposition of N_min_ was then calculated as follows:

$$Wet\;N\;\left(every\;rainfall\;event\;\left(kg\;N\;ha^{-1}\right)\right)=N_{min}concentration\;\left(mg\;N\;L^{-1}\right)\times precipitation\;\left(mm\right)\;\times 0.01$$ (3)

Atmospheric NO_2_ was collected with passive samplers using Gradko diffusion tubes from the ECN(UK Environmental Change Network website). The samplers were hung 1.5 m (at least 0.5 m higher than the canopy heights) above the ground and exposed between 20 days and one month in the air depending on the local NO_2_ concentration. The samples were measured by a colorimetric method at a wavelength of 542 nm.

NH_3_ samples were collected using Alpha passive samplers (Adapted Low-cost High Absorption; designed by the Center for Ecology and Hydrology, Edinburgh, UK). This equipment includes a tube, a plastic filter and a membrane (absorbed citric acid) and set at a height of around 1.5 m above the ground. The calculation equation is as follows:

V=D×A×t/L (4)

In this equation, *t* represents the time interval, *D* = 2.09×10^-5^m^-2^s^-1^ at 10° C,*A* = 3.463×10^-4^m^-2^, and L = 0.006m.

Airborne PM_10_ particles (particulate matter whose aerodynamic equivalent diameter is < 10 μm) were sampled using a medium flow particulate sampler (Tianhong, Wuhan, China) with a flow rate of 1.05 m^3^ min^-1^, and 7–10 daily samples of PM_10_ were collected at the research area.

### Data analysis

Nitrogen harvest index (NHI) is frequently used to assess N transport from shoots and leaves to the grain of a crop[3]. NHI of wheat and cotton was calculated as follows:

$$NHI=\frac{N_{grain}}{N_{aboveground-biomass}}$$ (5)

where *N*_grain_ and *N*_aboveground-biomass_ are N uptake of grain and crop biomass in a given treatment (CK, NPK, NPKS, NPKM or NPKM+).

Nitrogen mineralization was estimated from the balance of inputs and outputs in the control (N_0_) treatment according to the following formula:

$$N_{mineralization}=N_{CK\;uptake}+N_{residual\;soil\;Nmin}-N_{irrigation,\;deposition,seed/seedling}-N_{initial\;Nmin}$$ (6)

Where *N*_CK uptake_ is the N from the crop uptake of the control (CK), *N*_residual soil Nmin_ is the residual N in the top 100 cm of the soil profile after harvest; *N*_irrigation, deposition,seed/seedling_ is the N from deposition and irrigation; and *N*_initial Nmin_ is the N from the top 100 cm of the soil profile before sowing.

Apparent N losses were estimated after wheat and cotton and one cycle of the wheat-cotton rotation using the method proposed by Liu et al. [30]. The apparent N losses were calculated by differences between the inputs (fertilizer, initial soil mineral N, deposition, irrigation, seed/seedling, biological N fixation(BNF) and N mineralization) and outputs (uptake by crops and residual soil N_min_). The formula is as follows:

$$N_{apparent\;losses}=N_{rate}+\;N_{initial\;Nmin}+\;N_{environment}-\;N_{crop\;uptake}-\;N_{residual}$$ (7)

Where the N _rate_ is the application amount of N, the *N*_initial Nmin_and *N*_environment_ are the N from the fertilization and environment (irrigation, deposition N, and BNF)

All statistical analysis was performed with the SPSS 16.0 software package. Analysis of variance (ANOVA) was used to test for significance of treatments and means were compared by least significance difference (LSD) at the 5% level.

## Results

### N inputs from fertilizers and environment

Table 1 displays the N inputs of wheat and cotton under the two-year rotation experiment in the oasis cropland of northwest China. The average N input was 294 kg N ha^-1^ in the wheat season and 295 kg N ha^-1^ in the cotton season. The amounts of N input from rain and dry deposition were 35 and 31 kg N ha^-1^ yr^-1^ in wheat and cotton seasons, accounting for 12 and 10.6% of the total average N input. The irrigation water provided 8 kg N ha^-1^yr^-1^ input amount, accounting for 2.7% of the N input amount in the wheat season, and 17kg N ha^-1^ yr^-1^ input amount, accounting for 7% of the N input amount in the cotton season. The average environmental N (N from irrigation, rain and dry deposition) input is about 48.4 and 48.9 kg N ha^-1^ yr^-1^ for wheat and cotton, accounting for 20.0 and 20.2% of the total amount of N input, respectively. The N from seed is 5.2 and 0.9 kg N ha^-1^ yr^-1^ for wheat and cotton, accounting for 2.1 and 0.3% of the total average N input.

**Table 1:** Nitrogen inputs (kg N ha^-1^) in a wheat-cotton rotation in a field experiment in an extremely arid region of northwest China (2011–2012).

	Wheat season	Cotton season	System (wheat + cotton)
Fertilizer N^a^	246^b^	246	490
N from rainfall	28.9	25.7	54.6
N from dry deposition	6.3	5.3	11.6
N from Irrigation	8.0	17.0	25.0
N from seeds/seeding	5.2	0.9	6.1
Total input	293	294	587

^a^Comprises chemical fertilizer and organic manure.

^b^The conventional amount of fertilizer used locally (except CK and NPKM+).

### Mineral N accumulation and distribution after crop harvest

**Fig 4:**
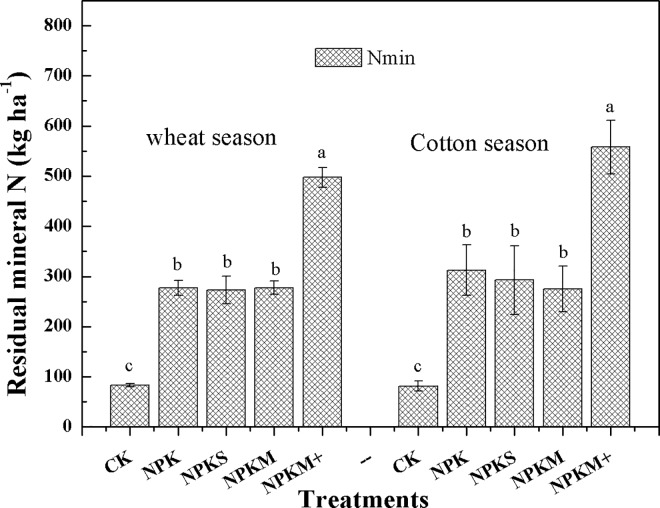
Residual mineral N from the top 100 cm of the soil profile after the crop harvests in our long-term experiment in an extremely arid zone of northwest China. The bars denote standard errors of the mean (n = 4).

Nearly all treatments (excluding the control) had a large N_min_ accumulation in the top 100cm of the soil profile, especially NPKM+ (497 and 558kg N ha^-1^after the wheat and cotton harvests (Fig 4). NPK and NPKS had moderately high N_min_ values, 278 and 273 kg N ha^-1^ after the wheat harvest and 313 and 293kg N ha^-1^ after the cotton harvest. NPKM had a lower N_min_ content (278 and 275kg N ha^-1^ after the wheat and cotton harvests, respectively) than the other treatments. Compared with NH_4_-N, NO_3_-N was dominant in all treatments except the control. The peak values of NO_3_-N appeared at 0–20 and 50'–70cm in the cotton season.

**Fig 5:**
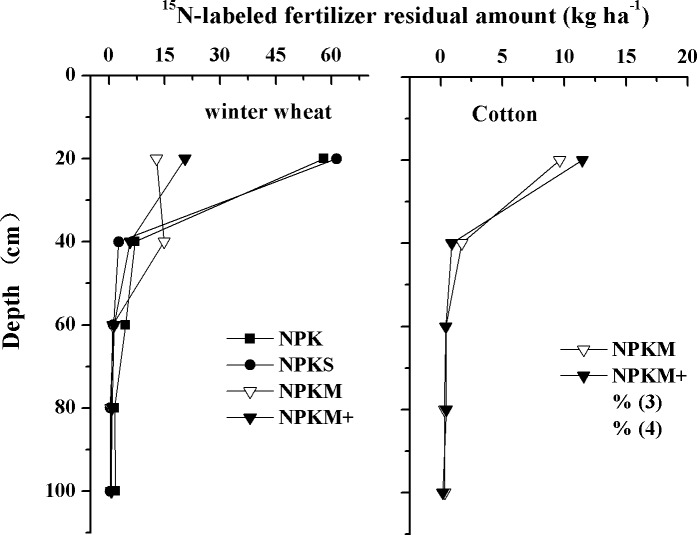
The leaching depth of ^15^N-labeled fertilizer in the top 1m of the soil profile in the wheat and cotton growing seasons.

In the two-year ^15^N-microplot experiment, chemical ^15^N accumulated mainly at 0–40cm soil depth in the wheat season and 0–60cm depth in the cotton season (Fig 5). In the wheat season we found that 95% of the ^15^N remained at 0–40cm soil depth in all N treatments. In the cotton season we found that 90% of the ^15^N accumulated at 0–60cm soil depth. This demonstrates that the water from the dripping irrigation method leached less than 60cm into the soil profile in both seasons.

### Crop yield and NHI

**Fig 6:**
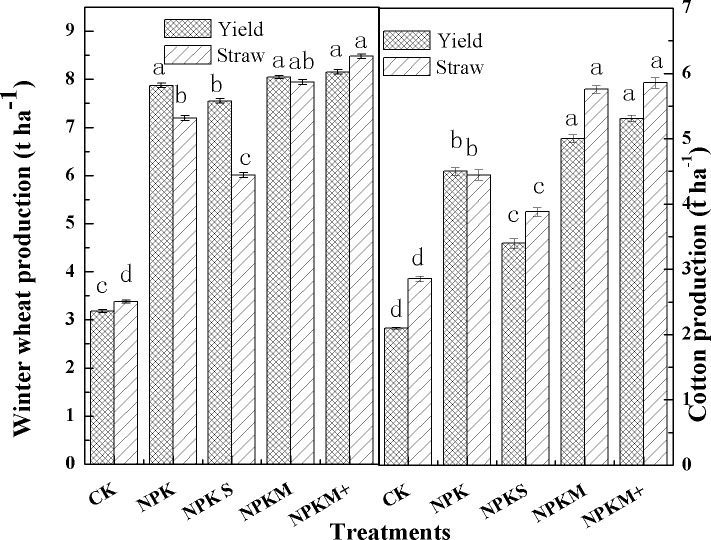
The yield and straw biomass of wheat and cotton from 2011 to 2012.

In the wheat season the yield followed the sequence CK< NPKS < other treatments (all p<0.05). Other treatments showed no significant difference in wheat production (p>0.05). Compared with other fertilizer-addition treatments, NPKM and NPKS treatments had the lowest straw production as shown in Fig 6, suggesting greater NHI of the two treatments (up to 0.75 and 0.82) (Table 2). In contrast, the NPKM+ treatment had a significant lower NHI than other treatments (p<0.01). In the cotton season NPKM and NPKM+ had the highest unginned cotton yields (p<0.05), with the NPK treatment second (4.5 t ha^-1^ yr^-1^), and NPKS had the lowest yield (3.4t ha^-1^ yr^-1^) which was significantly lower than other balanced fertilizer treatment in the cotton season (p<0.05). The NPKM and NPKS treatments had the highest NHI, NPKM+ was second with a value 0.52 and NPK had the lowest value (NHI 0.47).

**Table 2:** Crop N uptake, grain N level and NHI of wheat and cotton under different treatments in an extremely arid region of northwest China.

Treatment	Crop N uptake		Grain N level		N harvest index^b^	
	(kg ha^-1^)		(%)			
	Wheat	Cotton	Wheat	Cotton^a^	Wheat	Cotton
Control	67.5d	81.3d	1.6a	0.1a/2.3a	0.76b	0.49c
NPK	159.5b	172.3c	1.5b	0.1a/2a	0.74b	0.47c
NPKS	137.6c	186.1b	1.5b	0.07b/2b	0.82a	0.55a
NPKM	172.2a	207.2a	1.6a	0.1a/2.2b	0.75b	0.55a
NPKM+	174.1a	167.9c	1.5b	0.9a/1.9b	0.70c	0.52b

^a^The left side represents the cotton seed N level; the right side represents the cotton fiber N level.

^b^ Grain crop N uptake divided by aboveground N uptake.

Different letters denote significant difference by LSD at the 0.05 level.

In this two-year experiment suitable manure-addition treatments (NPKM) increased grain or unginned cotton yields in oasis arid croplands but NPK and NPKM+ did not further increase crop yields. Thus, at the same application rates of N, P, and K, combined application of inorganic fertilizers with compost did benefit the cotton yields and significantly increase the NHI under dripping irrigation and film mulching conditions.

### ^15^N balance

The ^15^N-labelled fertilizer experiment results are shown in Table 3. In the wheat season the NPKM treatment had the highest ^15^N-labelled fertilizer uptake rate and second was the NPK treatment. NPKM+ had the lowest ^15^N-labelled fertilizer recovery rate. NPKM had the highest ^15^N-residual rate in the soil profile, whereas NPK had the lowest ^15^N-residual rate in the soil profile. NPKM had the lowest loss of ^15^N-labelled fertilizer, accounting for 5% of total ^15^N-labelled fertilizer application, and NPK, NPKS and NPKM+ accounted for 28, 28.7 and 20.8%, respectively.

**Table 3:** The fate of ^15^N fertilizer in a wheat-cotton rotation system in a long-term field experiment in an extremely arid zone of northwest China.

Crop/Treatment		N rate	Crop uptake		Soil residual		Loss^a^	
		kg N ha^-1^	kg ^15^N ha^-1^	%	kg ^15^N ha^-1^	%	kg ^15^N ha^-1^	%
Wheat	Control	0						
	NPK	246	111.2a	45.2	65b	26.4	68.5a	28.4
	NPKS	246	89.8b	40.8	66.9b	30.4	63.2a	28.8
	NPKM	246	42.1d	51.3	39.8c	48.5	4.3c	0.2
	NPKM+	369	50.9c	31	71.1a	43.4	32b	25.6
Cotton	Control	0						
	NPK	246	102.5a	41.7	99.1a	40.3	43.4c	18
	NPKS	246	96b	43.6	61.9b	28.1	58.9b	28.3
	NPKM	246	38c	46.3	33.7cd	41.1	14.0d	12.6
	NPKM+	369	37c	22.6	40.5c	24.7	76.5a	46.6

Within each column, values with the same letter are not significantly different by LSD at the 0.05 level across soil depth.

^a^Portion unaccounted for.

Similar results were found in the cotton season. NPKM+ had the lowest ^15^N-labelled fertilizer recoveries, accounting for 24% of total ^15^N-labelled fertilizer application. No significant differences in ^15^N-labelled fertilizer recoveries occurred in NPK, NPKS and NPKM, accounting for 41.8, 43.8 and 44.5% of total ^15^N-labelled fertilizer application, respectively. NPKM had the highest ^15^N-labelled fertilizer residues and the lowest other losses. NPKS had relatively low ^15^N-labelled fertilizer rate soil residues. After both of the wheat and cotton harvests most of the residual ^15^N-labelled fertilizer remained at 0–60 cm soil depth irrespective of treatment.

### Apparent N losses

Apparent N losses from wheat and cotton including the crop rotation, accounting for all N inputs and outputs measured in this study, are displayed in Table 4. In the wheat season the initial N_min_ was 93, 242, 250, 276 or 434kg N ha^-1^for CK, NPK, NPKS, NPKM and NPKM+, respectively. NPKM+ was the treatment with the highest total N input.

**Table 4:** Apparent N balance of wheat-cotton rotation systems under different N management strategies (kg ha^-1^).

Treatment	N input					N output		Apparent N loss
	Chemical	Organic or Straw	CF+OF	Initial Nmin	Environmental N^a^	Crop uptake	Residual Nmin	(= input-output)
	Fertilizer (CF)	Fertilizer(OF)						
	kg N ha^-1^					kg N ha^-1^		kg N ha^-1^
*Wheat season*								
Control	0	0	0	93.3	48.4	67.5d	83.1	-8.9
NPK	246	0	246	242	48.4	159.5b	277	99.9
NPKS	220	26	246	250.4	48.4	137.6c	273	134.2
NPKM	82	164	246	275.6	48.4	172.2a	277.5	120.3
NPKM+	164	205	369	433.6	48.4	174.1a	497.4	179.5
*Cotton season*								
Control	0	0	0	83.1	48.9	81.3d	81.2	-30.5
NPK	246	0	246	277	48.9	172.3c	312.7	86.9
NPKS	220	26	246	273	48.9	186.1b	292.7	89.1
NPKM	82	164	246	277.5	48.9	207.2a	275.2	90
NPKM+	164	205	369	497.4	48.9	209.9c	557.7	147.7
*System*								
Control	0	0	0	93.3	97.3	148.8	99.1	-39.4
NPK	492	0	492	242	97.3	331.8	312.7	186.8
NPKS	440	52	492	250.4	97.3	323.7	292.7	223.3
NPKM	164	328	492	300.3	97.3	379.4	275.2	210.3
NPKM+	328	410	738	433.6	97.3	342	557.7	327.2

^a^ N from irrigation, dry and wet N deposition, seeds/seeding and biological N_2_ fixation (see Table 1).

**Fig 7:**
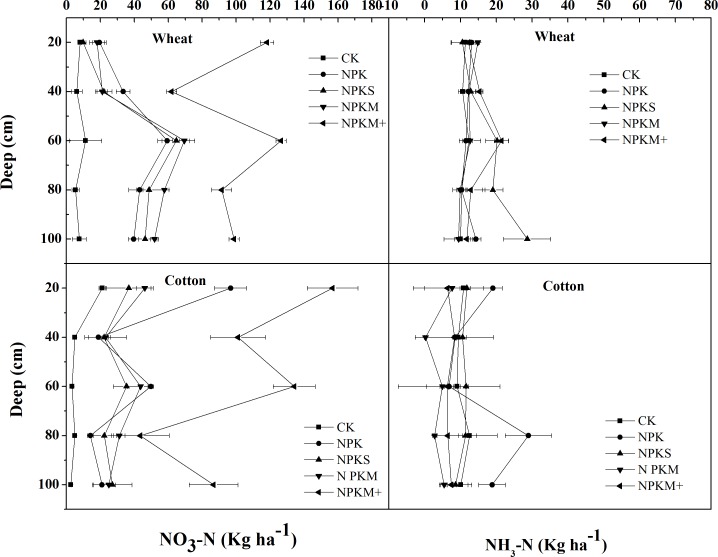
Distribution of NO_3_^-^–N and NH4^+^-N in the soil after the wheat and cotton harvests in the field plot experiment in northwest China. The bars denote standard errors of the mean, n = 3.

After the wheat harvest, nearly all the fertilizer treatments had significant residual N_min_ increases in the top 1m of the soil profile except for the NPKM and the control (slightly increase or decrease). The values were 83.1, 277, 273, 278 and 497 kg N ha^-1^for CK, NPK, NPKS, NPKM and NPKM+, respectively. After the cotton harvest the N_min_ in NPK, NPKS and NPKM+ at 1m soil depth showed a significant increase, especially in NPKM+ and NPK with increased values of 35.7 and 60.3 kg ha^-1^, respectively. The N_min_ in NPKM did not increase and was almost in balance, and the control showed a declining trend as shown in Fig 7.

In addition, nearly all the treatments had relatively high apparent N losses, especially NPKM+ in which the value reached 180 kg N ha^-1^yr^-1^ in the wheat season and 148kg N ha^-1^yr^-1^, accounting for 37.3 and 30.9% of total N input. NPK had the lowest apparent N loss (99.9kg N ha^-1^yr^-1^in the wheat season and 86.9kg N ha^-1^yr^-1^ in the cotton season), accounting for 30.3 and 26.3% of total N input. NPKM and NPKS had relatively high apparent N losses with values of 120 and 134 kg N ha^-1^yr^-1^ in the wheat season and 90 and 89 kg N ha^-1^yr^-1^ in the cotton season. Across the whole system, NPKM+ was the treatment with the highest soil residual N_min_(558 kg N ha^-1^) and highest apparent N loss (369 kg N ha^-1^). NPKM had lower amounts of soil residual N_min_(275 kg N ha^-1^) than NPK or NPKS but a slightly higher apparent N loss (245 kg N ha^-1^). However, if the control is excluded the total N inputs of all the treatments were very large.

## Discussion

### Nitrogen input

As in other climatic zones, chemical fertilizers, manures and straw-return in extreme arid croplands are the most frequently used agricultural inputs, accounting for the majority of N inputs. In our study area dry and wet N deposition (33 kg N ha^-1^ yr^-1^) represent an important source of environmental N due to increasing use of fossil energy and urbanization. Due to high evaporation and demand for water, the large amounts of irrigation water used are pumped from deep groundwater supplies to the surface and this brings relatively large amounts of NO_3_-N or NH_4_-N into the cropping area. We estimate total environmental N sources of up to 48.5 kg N ha^-1^ yr^-1^(N deposition, irrigation water and seed) in our research area making this an important source of N inputs.

Most of the treatments that we examined in the field experiment had large amounts of mineral N in the soil profile. The initial N_min_ value ranged from 240 to 447 kg N ha^-1^ to a depth of 1 min the soil profile and this was significantly higher than reported at other experimental sites [3, 8, 31]. For example, in paddy soils in southwest China the cumulative soil mineral N ranged from 42 to 115 kg N ha^-1^yr^-1^ in commercial fields. The very high soil residual mineral N contents found in the present study may be mainly attributable to the absence of leaching below a depth of 1 m and ammonia volatilization. The nitrogen use efficiency may have increased under film mulching which has been shown in numerous studies to greatly reduce ammonia volatilization [32–35].

Liu et al. [26]found that total NH_3_-N volatilization in non-mulched fields (30–34 kg ha^-1^) was ten times that in mulched fields during the first two months after urea application, and drip irrigation can also reduce N_min_ leaching losses by maintaining the soil water content to a depth of 1 m in the soil profile. In addition, the conventional fertilizer N are relatively high, ranging from 220 to 300 kg N ha^-1^yr^-1^ in the oasis grey desert agricultural land[21]. Moreover, the large differences between day and night temperatures and clear alternate freezing and thawing cycles (in winter from November to March) may stimulate the soil microbial community to produce soil mineral N and N_2_O by increased degradation of organic matter. Lv et al. [17]found a sharp increase in mineral N after the freezing and thawing stage in grey desert croplands. As a consequence, high concentrations of residual N inevitably occur in arid agricultural fields.

### Yield, NHI and NUE

Oasis agriculture relies greatly on irrigation and fertilization to increase or maintain crop yields on relatively infertile soils. Numerous studies have demonstrated that improved desert farmlands can significantly increase crop yields. For example, wheat yields of 7.6–8.2 t ha^-1^ can be achieved at our research area, and yields similar to those in non-arid areas can be obtained [5]. Cotton production can reach 3.4–5.3 t ha^-1^, higher than in many non-arid areas, and Xinjiang has therefore become one of the largest cotton producing areas in China. The high light intensities and temperatures increase photosynthetic potential and the increasing use of mulching film and drip irrigation technology are also important factors leading to higher fertilizer and water use efficiency. In our research area the NHI remained at 0.70–0.82, a range of values higher than in semi-arid regions where the wheat NHI ranges from 0.48 to 074, but slightly lower than the North China Plain wheat NHI (0.73–0.83) although there are a number of factors involved (the crop genotype, environmental conditions, soil type and fertilization methods). Cotton has a low NHI (0.38–0.55) compared with other crop species because the N content of cotton lint is relatively low and that of the straw is relatively high compared with wheat straw. In addition, our yields in the balanced treatment showed no significant difference in the wheat season but were higher in the NPKM treatment in the cotton season due to a higher N absorption efficiency (grain or fiber). The NPKS treatment also gave high cotton yields with high N absorption efficiency but no corresponding advantage during the wheat season. The NHI of different treatments also showed significantly differences. NPKS and NPKM treatments had significantly higher NHI, indicating that the straw and an appropriate amount of organic fertilizer were beneficial by increasing the grain or fiber production due to enhancement of soil physicochemical properties and microbial activity.

The mulching film and drip irrigation also improve the crop N use efficiency (NUE) in our research area. For example, the NUE in the balanced fertilizer treatment achieved 40.8–51.3% during the wheat season and 41.7–46.3% in the cotton season, and reached higher levels than the average values across China[5], indicating that agricultural management in arid desert oasis areas can greatly enhance the NUE. In our experiment the manure or straw amendments gave higher NUE values and indeed increased both crop yields and NUE, a finding that is consistent with studies in other climatic zones.

### Nitrogen outputs and apparent N losses

Fertilizer application rates in arid grey desert croplands have decreased substantially from 500–700 to 230–350 kg N ha^-1^yr^-1^[36]. This has been possible mainly due to improvements in agricultural management practices such as the widespread use of drip irrigation and mulching film which have greatly enhanced the efficiency of utilization of fertilizer N. However, in our two-year field experiment we found that NPKM+ had the highest residual N_min_ in both crop seasons due to excessive fertilizer inputs. The balanced fertilizer treatments also resulted in high soil residual N_min_, especially the NPK treatment with an increase of 35 kg N ha^-1^ in the wheat season and 35.7 kg N ha^-1^in the cotton season. The NPKS treatment showed an increase of 22.6kg N ha^-1^ in winter and 19.7kg N ha^-1^. However, in the NPKM treatment the accumulation N_min_ was lower than in other balanced treatments and our experiment indicates that overuse of chemical fertilizer N does occur but appropriate manure amendment can reduce the residual N in the soil profile due to higher crop N uptake.

In addition, we found that apparent N losses were higher in our research area than other locations, including the NPKM treatment. For example, the apparent N loss is usually < 100 kg N ha^-1^ in optimized N treatment on the North China Plain [30,37]. In the present study the apparent N loss ranged from 100 to 180 kg N ha^-1^ in the wheat season and 52 to 148 kg N ha^-1^ in the cotton season. This may be explained firstly by excessive fertilizer inputs. Conventional chemical fertilizer N application rates in our research area can reach 246 kg N despite a general decline in N application rates. Xu et al. [28] and Dong et al. [38] found N_min_ at a depth of 3 m down the soil profile due to leaching from the very high levels of precipitation in winter and historical overuse of fertilizer N. Secondly, drip irrigation and film mulching style have changed the pattern of movement of N, with more inorganic fertilizer N remaining at the soil surface due to the higher amounts of water in the top 1m of the soil profile. Thirdly, extremely arid croplands are subject to substantial drying and wetting and freezing and thawing cycles than other locations due to the high frequency of irrigation and longer winters (November to April). Numerous studies have demonstrated that alternating drying and wetting and freezing and thawing can accelerate the mineralization of soil organic matter [39–40]. This could lead an increase in N_min_ derived from the mineralization of soil organic matter.

Fertilizer N management in oasis desert croplands therefore requires further refinement. Full consideration of environmental N inputs must be included to determine the fertilizer N application rates used and more efficient application strategies must be developed to further reduce the levels of soil residual N_min_ and the apparent N losses must be taken into account in future studies.

## Conclusions

In the present study the N inputs from atmospheric dry and wet deposition accounted for 31–35 kg N ha^-1^yr^-1^ and those from irrigation water accounted for 8–17kg N ha^-1^ yr^-1^, indicating that abundant environmental N enters the cropping system in this region. Although mulch film and drip irrigation technology are now widespread in the region, most treatments had high initial Nmin concentrations in the top1m of the soil profile. From the wheat season to cotton, NPKM+ showed a significant increase in soil Nmin, followed by NPK and NPKS, and NPKM was close to maintaining Nmin in balance during the two years of the experiment. In the wheat season, in addition to the significantly higher apparent N loss in NPKM+, NPKS and NPKM also showed relatively high apparent N losses, and NPK had the lowest apparent N loss. In the cotton season, despite the high apparent N loss in NPKM+, the balanced fertilizer treatment showed no significant difference. Overall, the overuse of fertilizer N and the failure to fully take into account the high inputs of environmental N resulted in excessive accumulation of N in this extremely arid cropland area. It will be necessary to reconsider the management of N inputs in the future, especially those derived from irrigation and dry and wet deposition. It is also necessary to further refine the application rates of manure and fertilizer combinations simultaneously.
